# Genetic Subtyping, Biofilm-Forming Ability and Biocide Susceptibility of *Listeria monocytogenes* Strains Isolated from a Ready-to-Eat Food Industry

**DOI:** 10.3390/antibiotics9070416

**Published:** 2020-07-16

**Authors:** Joana Catarina Andrade, António Lopes João, Carlos de Sousa Alonso, António Salvador Barreto, Ana Rita Henriques

**Affiliations:** 1 CIISA–Centre for Interdisciplinary Research in Animal Health, Faculty of Veterinary Medicine, University of Lisbon, 1300-477 Lisbon, Portugal; ajoanacatarina@gmail.com (J.C.A.); asbarreto@fmv.ulisboa.pt (A.S.B.); 2 Laboratório de Bromatologia e Defesa Biológica do Exército, 1849-012 Lisbon, Portugal; joao.aebl@exercito.pt (A.L.J.); alonso.ces@exercito.pt (C.d.S.A.)

**Keywords:** biocide, *Listeria monocytogenes*, biofilm, planktonic culture, pulsed-field gel electrophoresis

## Abstract

*Listeria monocytogenes* is a foodborne pathogen of special concern for ready-to-eat food producers. The control of its presence is a critical step in which food-grade sanitizers play an essential role. *L. monocytogenes* is believed to persist in food processing environments in biofilms, exhibiting less susceptibility to sanitizers than planktonic cells. This study aimed to test the susceptibility of *L. monocytogenes* in planktonic culture and biofilm to three commercial food-grade sanitizers and to benzalkonium chloride; together with the genetic subtyping of the isolates. *L. monocytogenes* isolates were collected from raw materials, final products and food-contact surfaces during a 6-year period from a ready-to-eat meat-producing food industry and genetically characterized. Serogrouping and pulsed-field gel electrophoresis (PFGE) revealed genetic variability and differentiated *L. monocytogenes* isolates in three clusters. The biofilm-forming ability assay revealed that the isolates were weak biofilm producers. *L. monocytogenes* strains were susceptible both in the planktonic and biofilm form to oxidizing and ethanol-based compounds and to benzalkonium chloride, but not to quaternary ammonium compound. A positive association of biofilm-forming ability and LD_90_ values for quaternary ammonium compound and benzalkonium chloride was found. This study highlights the need for preventive measures improvement and for a conscious selection and use of sanitizers in food-related environments to control *Listeria monocytogenes.*

## 1. Introduction

*Listeria monocytogenes* is an ubiquitous small Gram-positive bacterium widespread in the natural environment [1]. It is also an opportunistic pathogen responsible for human listeriosis, a severe disease with high hospitalization and case fatality rates [2,3]. Its psychrotrophic nature and the ability to survive and multiply under extreme physicochemical conditions [4] may explain the difficulty of controlling its presence in refrigerated environments [5]. 

This pathogen is often associated to ready-to-eat (RTE) food products, with contamination occurring during food processing production [6,7]. Incoming raw materials, food handlers, and even processed ingredients and products are frequent sources of *L. monocytogenes* contamination [8]. After entering a food producing facility, *L. monocytogenes* can become a long-term resident, being able to persist for months or years within the premises, including food contact equipments [9]. Once established, *L. monocytogenes* biofilms can persist, resulting in the potential continuous contamination of the food products [10].

*L. monocytogenes* has the ability to adhere to different surfaces within the food industry, such as plastic, rubber, stainless steel, glass, and produce biofilms [5,11]. Biofilm formation is affected by many factors, such as strain-specific properties, composition of the attachment surface, and environmental conditions [12]. Previous works relating *L. monocytogenes* serotypes and biofilm formation remained inconclusive, although several authors have addressed it [13,14,15,16]. 

In the biofilm, bacteria are embedded by an extracellular matrix able to function as a structural scaffold and protective barrier against various stresses and antimicrobials, like those encountered in the food processing environment [13,17]. Biofilms are associated to increased resistance to sanitizing compounds, due to bacterial exposure to sublethal biocide concentrations, acquiring resistance to antimicrobials over time [17,18].

The validation of sanitizers is essential to avoid the misuse of biocides that may end-up promoting resistance of *L. monocytogenes* virulent strains. Still, the effectiveness of commercial food-grade sanitizers is tested on planktonic microorganisms, but the biofilm environment may change the response of every strain involved [19]. Among food-grade sanitizers used in RTE food processing premises, oxidizing disinfectants and quaternary ammonium compounds are the most popular, due to their broad-spectrum activity against bacteria, high efficacy and low cost [20,21]. Nevertheless, *L. monocytogenes* resistance to these compounds has been described, whether in planktonic cultures or in biofilms [10,22]. The same was reported for benzalkonium chloride, a quaternary ammonium compound [23,24,25].

In this work, the susceptibility of *L. monocytogenes* in planktonic culture and biofilm to three commercial food-grade sanitizers and to benzalkonium chloride was assessed. For that, *L. monocytogenes* isolates collected from a RTE food-producing industry during a 6-year period were genetically characterized and their biofilm-forming ability was assessed, prior to biocide susceptibility testing.

## 2. Results and Discussion

### 2.1. Characterization of L. monocytogenes Isolates Collection

The overall proportion of positive samples (food and food related environment) contaminated by *L. monocytogenes* was 26.3% (20/76) (Table 1). This high percentage is in line with other studies in Portugal [26] that reported 25% of positive samples in ham, 11.1% in blood sausage and 2.3% in dry cured ham collected from producers and retailers.

### 2.2. L. monocytogenes Confirmation and Serogrouping by PCR

All of the *L. monocytogenes* presumptive isolates (*n* = 20) obtained by conventional microbiological methods belonged to the *Listeria* genus, but only 17 were confirmed as *L. monocytogenes* by PCR [27]. Among these 17 isolates, four different molecular serogroups were identified (Table 2).

Most of the isolates belonged to serogroup IIc (52.9%), followed by serogroup IIa (35.3%), IIb and IVb (each with 5.9%). In line with our results, other authors have reported similar findings. Lotfollahi et al. [28] found serogroup IIc to be the most prevalent in *L. monocytogenes* isolates from several foods retailed in Iranian markets. In another study, Montero et al. [29] found serogroup IIa to be the most common one in RTE meat-based products collected from different retail stores and industrial processing plants in Santiago, Chile, although serogroup IIb, IIc and IVb strains were also present. In an investigation assessing serogroup diversity of *L. monocytogenes* isolates in food from central and northern regions in Italy, 67.5% of isolates belonged to serogroup IIa [30]. Rodríguez-López et al. [31] reported similar results in samples collected from different food-related premises in Northwest Spain during 2010 and 2011, of which only 5.9% of isolates belonged to serogroup IVb. Torresi et al. [32] reported a predominance of serogroup IIa and IIc strains in several different cheeses in Italy.

Molecular serotyping is a rapid and useful method for first-level characterization of *L. monocytogenes* [16]. Still, to allow for a more reliable characterization of strains and contamination routes investigation, other molecular subtyping methods, such as pulsed-field gel electrophoresis (PFGE) should be used [33].

### 2.3. Pulsed-Field Gel Electrophoresis Typing

**Figure 1:**
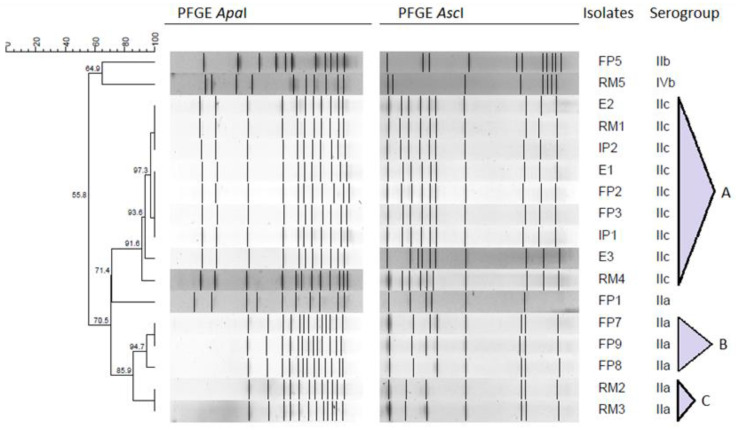
Dendrogram of the *Apa*I-*Asc*I PFGE profiles and corresponding serogroup for 17 *L. monocytogenes* selected isolates.

Figure 1 presents the resulting dendrogram of 17 *L. monocytogenes* strains considering *Apa*I and *Asc*I restriction patterns and serogroups. Pulsotypes were considered to be clones when they had at least 90% of similarity.

The different food and environment samples presented six PFGE types. Three clusters were identified (indicated as A, B and C in Figure 1), while FP5, RM5 and FP1 pulsotypes had a distinct PFGE profile. 

The first cluster (Figure 1, cluster A) includes 9 strains, corresponding to 52.9% of all the analyzed isolates. These strains with identical restriction patterns and exhibiting the same serogroup (serogroup IIc) were collected from raw materials, intermediate products, finished products, and food-contact surfaces in a time frame of 14 months (from February 2013 to April 2014). When comparing cluster A food-contact surfaces and finished products strains’ profiles, results suggest the possibility of a common source. It is noteworthy that *L. monocytogenes* strain RM4 collected in 2014 has 91.6% similarity with strains collected in 2013. This is suggestive of a potential persistent contamination within the food industry, although more studies should be considered in order to establish source attribution. Kurpas et al. [34] linked *L. monocytogenes* presence in food processing environments, such as abattoirs, RTE meat-processing industries and retail establishments to cross-contamination. 

Cluster B includes three strains (FP7, FP8 and FP9) collected from different finished products between May 2014 and April 2015, all belonging to serogroup IIa. *L. monocytogenes* strain FP7 shares an indistinguishable profile with strain FP9, collected 1 year later. Apart from being suggestive of persistence over time, which might be due to *L. monocytogenes* survival and growth in niches within the food environment, these strains belong to serogroup IIa, which is the one most commonly associated to food-related environments [1,30]. 

Cluster C includes 2 strains (RM2 and RM3) collected between 2013 and 2014, from different raw materials. As seen before in cluster A, *L. monocytogenes* pulsotypes identified in raw materials exhibit high similarity with pulsotypes from equipment and finished products. These pulsotypes may persist due to the repeated re-introduction of strains from the external environment into food processing facilities over time [35]. Suppliers should be addressed to understand the origin of some strains, although results underline cross-contamination as a possible way of disseminating *L. monocytogenes* in the assessed food industry. A strict selection and control of suppliers seems to be a preventive measure of upmost importance [36]. Three distinct pulsotypes can also be seen in the resulting dendrogram. FP5 and RM5 strains were collected 1 year apart from each other and presented distinct pulsotypes (64.9% of similarity), belonging to serogroups IIb and IVb, respectively. FP1 isolate exhibits a different PFGE profile from other serogroup IIa strains (71.4% of similarity), which might be due to the fact that serogroup IIa includes atypical strains [27,37]. 

The presence of serogroups IIa, IIb and IVb isolates suggests a potential public health hazard associated with these RTE meat-based products consumption, since these are the serogroups more commonly associated to human infection [38,39]. 

### 2.4. Biofilm Formation Assay

After serogrouping and PFGE typing, 10 *L. monocytogenes* strains were selected for the biofilm formation assay in order to have representatives with different profiles (serogroups and pulsotypes). *L. monocytogenes* CECT 4031, CECT 911, CECT 935, and CECT 937 were also included in order to investigate differences between strains of different serogroups. 

**Figure 2:**
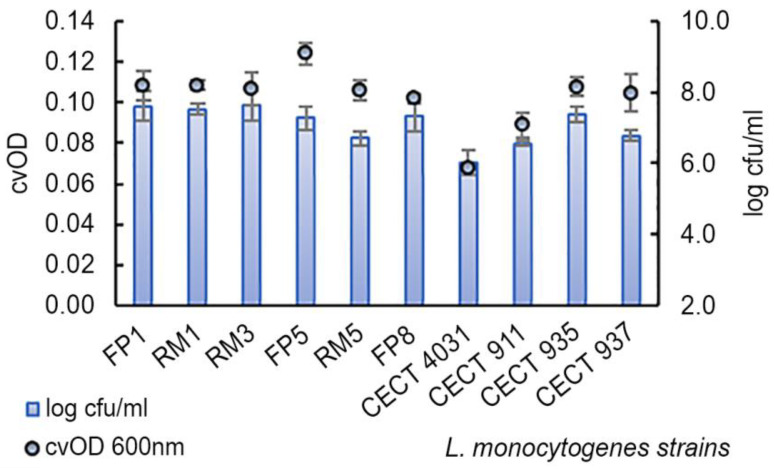
Average and standard deviation of log cfu/mL and cvOD of 5-day *L. monocytogenes* biofilms.

The assessed strains in biofilms revealed cvOD values ranging from 0.068 ± 0.001 to 0.1240 ± 0.006 and viable cell counts of 6.0 ± 0.4 log cfu/mL to 7.6 ± 0.4 log cfu/mL after 5 days of growth in polystyrene microtiter wells (Figure 2). 

According to Stepanović et al. [40] classification, all the strains (*n* = 10) revealed a weak biofilm-forming ability. Similar results were obtained by Meloni et al. [41] when studying *L. monocytogenes* isolates from fermented sausage processing plants: 65% of all isolates were weak biofilm producers. However, in our work, the assessed strains exhibited significantly different degrees of biofilm-forming ability based on cvOD values (*p* = 0.0066), and VCC results did not reflect the same biofilm-forming ability classes as those obtained using cvOD values. Considering VCC values, all the strains, except *L. monocytogenes* FP1 and RM3 isolates, revealed lower values than *L. monocytogenes* CECT 935, which exhibited 7.4 ± 0.2 log cfu/mL. On the other hand, when considering cvOD values, *L. monocytogenes* FP1, RM1, and FP6 isolates exhibited higher cvOD values than reference *L. monocytogenes* CECT 935 (0.1078 ± 0.005). Considering cvOD and VCC values, *L. monocytogenes* CECT 4031 revealed the lowest values for both parameters at 30 °C. The obtained difference between these two parameters is due to the nature of each method of determination. While cvOD measures the turbidity of a suspension and quantifies total biomass (viable and non-viable cells, but also extracellular matrix components), VCC only considers live cells [42]. Taking into account the selected methods to analyze biofilm formation-VCC (log cfu/mL) and cvOD, Pearson’s correlation analysis was performed. According to Pearson’s correlation coefficient (*ρ* = 0.7749, *p* = 0.009), there is a positive and strong correlation between both parameters, which indicates that both methods present a good relationship, being reliable to quantifying *L. monocytogenes* biofilm formation, complementing each other. 

When relating the biofilm-forming ability using cvOD values with the assessed *L. monocytogenes* serogroups, no significant differences were found (*p* = 0.526) and the same happened for VCC values (*p* = 0.929) (Table 3). 

Similar results were obtained by Di Bonaventura et al. [43] when studying the association of phylogeny and biofilm production. Nevertheless, this study’s results counteract the ones obtained by Meloni et al. [41], in which serotypes 1/2a, 1/2b and 4b isolates presented a higher adherence when compared to serotype 1/2c isolates. 

Other authors have shown that *L. monocytogenes* strains from different sources and serogroups are able to produce biofilms on a variety of surfaces, depending on the strain, surface and culture conditions [13,44]. Previous works reported that *L. monocytogenes* strains varied significantly in their ability to produce biofilm, but no trends could be observed when isolates’ serotype and source were compared [3,40]. It is important to highlight that since there is a link between virulence and *L. monocytogenes* serotype, a continuous discussion relating biofilm formation and serotypes goes on, in order to determine whether biofilm formation is related to disease incidence [1,14]. 

For further testing, five *L. monocytogenes* strains (RM1, RM3, RM5, CECT 4031, and CECT 935) were selected based on serogrouping and biofilm formation parameters data analyses.

### 2.5. Biocides Activity Testing Assay

#### 2.5.1. Activity towards *L. monocytogenes* Planktonic Suspension

The effect of food-grade commercial sanitizers, including an oxidizing compound (OxC), a quaternary ammonium compound (QaC) and an ethanol-based compound (EthC) on the selected five *L. monocytogenes* strains was assessed. Tested concentrations were selected based on the manufacturer’s recommendation for use in food contact surfaces. The manufacturer’s recommended concentrations for OxC and EthC were found to be equally effective in inactivating the five tested strains in planktonic suspension, although this was not observed for QaC. 

*L. monocytogenes* planktonic cells were inactivated by 50 ppm or more (100 and 150 ppm) of OxC. Norwood and Gilmour [45] reported that a 30 sec exposure to 10 ppm free chlorine was enough to completely eliminate planktonic *L. monocytogenes* culture. 

*L. monocytogenes* strains were exposed to increasing concentrations of EthC (50%, 70%, and 100%) that seemed to be effective in inactivating planktonic cells. Similar results were obtained by Aarnisalo et al. [46]. 

**Figure 3:**
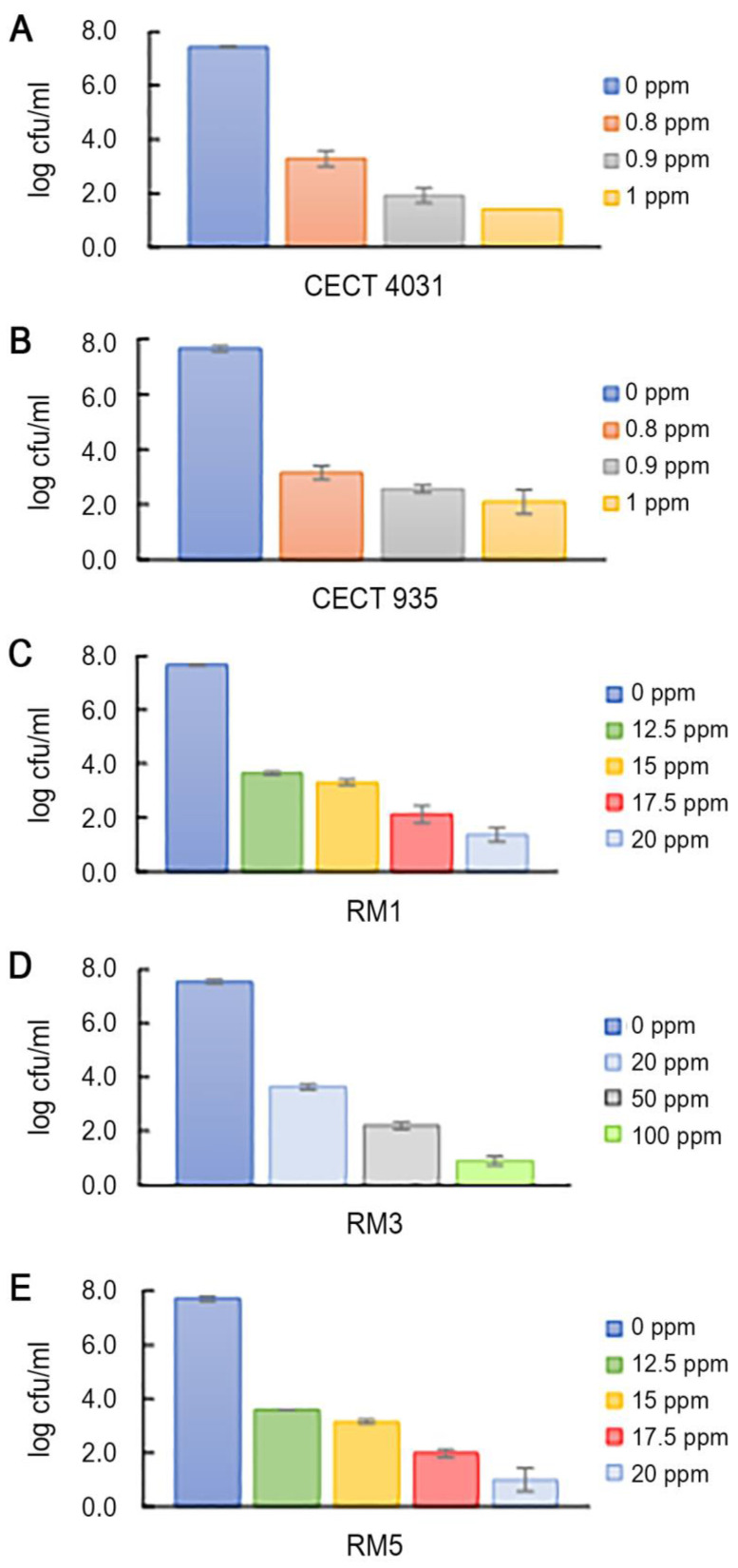
Viable cell counts average and standard deviation (error bars) of the tested planktonic *L. monocytogenes* strains after treatment with BaC. (**A**) *L. monocytogenes* CECT 4031; (**B**) *L. monocytogenes* CECT 935; (**C**) *L. monocytogenes* RM1; (**D**) *L. monocytogenes* RM3; (**E**) *L. monocytogenes* RM5.

*L. monocytogenes* planktonic forms enumeration after QaC treatment was not possible to perform within the tested concentration range. Some authors have reported resistance to QaCs in *L. monocytogenes* strains [47,48,49] and active efflux pumps are considered the main mechanism for *L. monocytogenes* tolerance to QaCs [50]. Because it was not possible to determine *L. monocytogenes* susceptibility to QaC, benzalkonium chloride (BaC) was used to evaluate *L. monocytogenes* planktonic cells susceptibility. Figure 3 presents the effects of BaC treatment on the five selected *L. monocytogenes* strains planktonic suspensions. As shown, all strains in the planktonic form presented different susceptibilities to BaC, being affected by different concentrations. Reference strains *L. monocytogenes* CECT 4031 and CECT 935 were the most susceptible, presenting more than 4-log cfu/mL reduction when exposed to 0.8 ppm of BaC. *L. monocytogenes* RM1, RM3 and RM5 strains were less susceptible, presenting 4-log cfu/mL reduction only for concentrations higher than 12.5 ppm for RM1 and RM5 and 20 ppm for RM3. To have an 8-log cfu/mL reduction, *L. monocytogenes* CECT 4031 and CECT 935 planktonic cells were exposed to 2 ppm of BaC. The same was observed when RM1 and RM5 and RM3 were subjected to 25 ppm and 150 ppm, respectively. In line with our results, Nocker et al. [51] reported that the exposure of *L. monocytogenes* strains to BaC concentrations higher than 30 ppm for 30 min was able to reduce bacterial colonies as measured by plate counts. 

#### 2.5.2. Activity towards *L. monocytogenes* 5-day-old Biofilms

**Figure 4:**
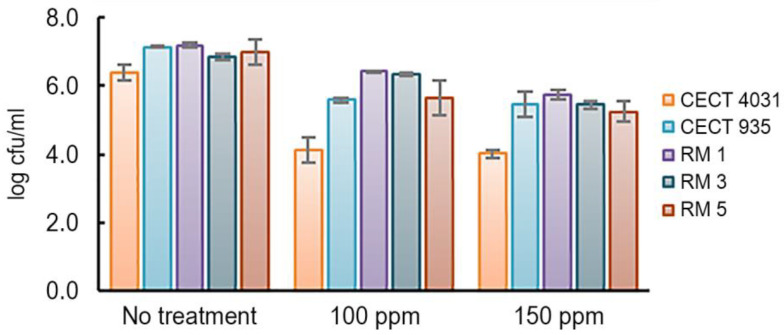
Average and standard deviation (error bars) of tested *L. monocytogenes* 5-day-old biofilms’ VCC after QaC treatment.

The biocide activity testing assay on biofilms was based on the enumeration of viable cells. The three commercial compounds were tested on 5-day-old biofilms according to the manufacturer’s recommended concentrations. As was observed for *L. monocytogenes* planktonic cells, both OxC and EthC tested concentrations, which were within the manufacturer’s recommended concentrations, were able to eliminate biofilms of all the tested isolates in 5 min at 20 °C. In fact, it was reported that 200 ppm of sodium hypochlorite, an OxC, is enough to eliminate at least 20% of *L. monocytogenes* biofilms [3,45]. In contrast, after QaC’s treatment, no susceptibility to this biocide was observed. Figure 4 presents the effect on VCC after treatment with QaC on the selected *L. monocytogenes* 5-day-old biofilms. 

In general, QaC was not effective in removing *L. monocytogenes* 5-day-old biofilms. As shown in Figure 4, when exposed to 150 ppm of QaC, *L. monocytogenes* CECT 4031 presented the highest reduction (from 6.4 log cfu/mL to 4.0 log cfu/mL). The remaining *L. monocytogenes* strains presented approximately 1-log cfu/mL reduction in VCC values. QaC resistance in *L. monocytogenes* biofilms has been reported [52,53]. Taking into account that this biocide is commonly used in food-related environments, these results are worrisome, as *L. monocytogenes* biofilms present a potential risk in food safety [54]. 

**Figure 5:**
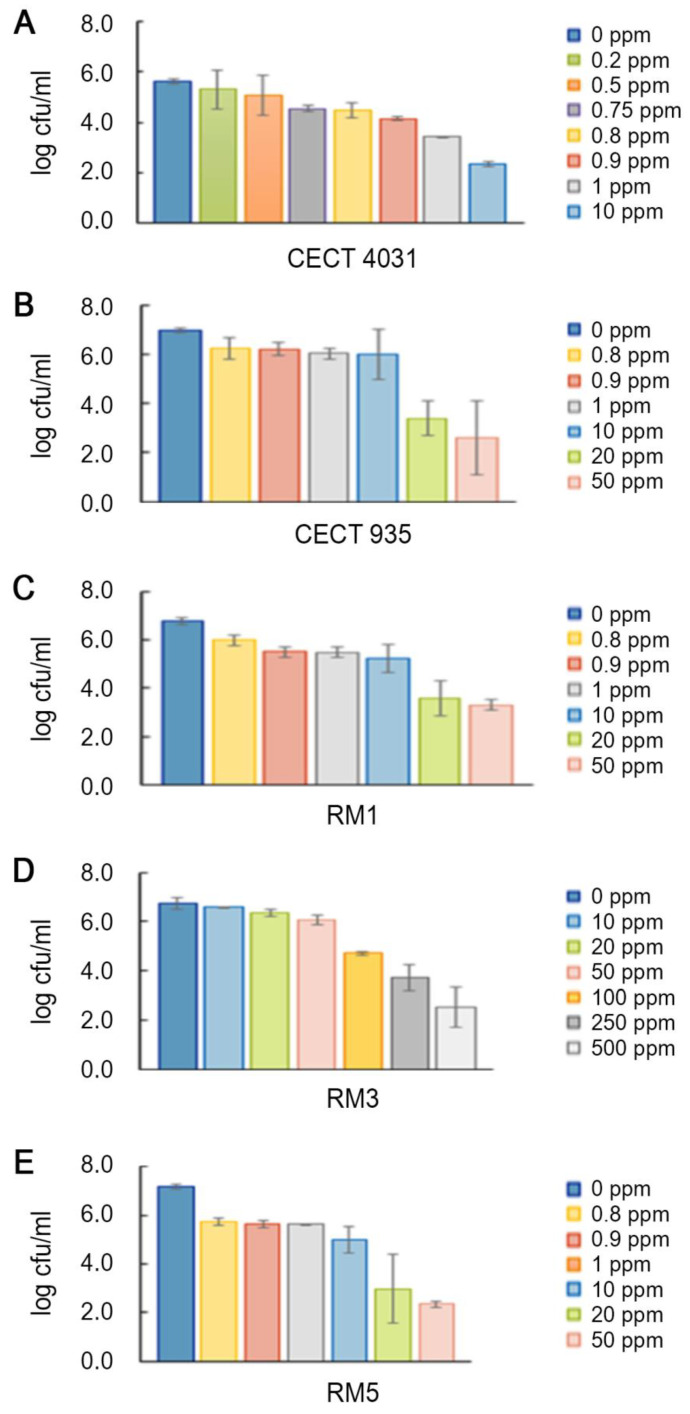
Viable cell counts average and standard deviation (error bars) of the tested *L. monocytogenes* 5-day-old biofilms after BaC treatment. (**A**) *L. monocytogenes* CECT 4031; (**B**) *L. monocytogenes* CECT 935; (**C**) *L. monocytogenes* RM1; (**D**) *L. monocytogenes* RM3; (**E**) *L. monocytogenes* RM5.

Figure 5 presents the tested concentration range of BaC’s in *L. monocytogenes* 5-day-old biofilms. In general, *L. monocytogenes* 5-day-old biofilms’ VCC were affected by different BaC concentrations, as happened for planktonic suspensions. While *L. monocytogenes* CECT 4031 was the most susceptible to BaC’s treatment and also presented the lowest biofilm-forming ability, *L. monocytogenes* RM3 strain was the less susceptible, but presented the highest biofilm-forming ability based on VCC values. A 3-log cfu/mL reduction was observed for *L. monocytogenes* CECT 4031 after 5 min of exposure to 10 ppm of BaC. On the other hand, for a similar reduction on *L. monocytogenes* RM3 biofilm, 250 ppm of BaC were necessary. Comparing these results to those obtained for planktonic cells, it seems that *L. monocytogenes* biofilms are less susceptible to BaC’s tested concentrations, since a higher BaC’s concentration is needed to have an equivalent log cfu/mL reduction. 

One example is *L. monocytogenes* RM3 isolate that in biofilm presented a 2-log cfu/mL reduction when exposed to 100 ppm of BaC and a 3-log cfu/mL reduction when exposed to 250 ppm, while the exposure to 150 ppm of BaC in the planktonic form was enough to cause a 8-log cfu/mL reduction. It has been previously discussed that in biofilm form, *L. monocytogenes* is more resistant to stress and sanitizing agents than planktonic cells [41,55]. Nakamura et al. [54], when assessing the sanitizing effect of BaC in *L. monocytogenes* planktonic cells and biofilms, reported that biofilm formation and tolerance to BaC might be related. Tolerance to BaC has also been reported by Piercey et al. [23] after testing BaC resistance and susceptibility based on the minimum inhibitory concentration, and by Xu et al. [24] after investigating phenotypic and genotypic tolerance to BaC based on susceptibility testing and molecular methods. Although in the last years several studies have focused on biofilm elimination, possible facilitating strategies are still unclear. 

**Figure 6:**
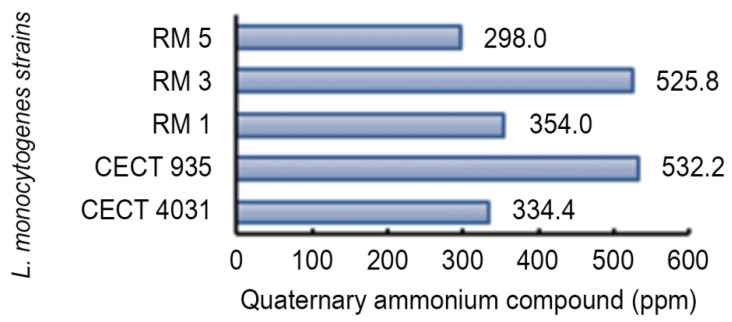
LD_90_ estimated values of *L. monocytogenes* tested strains in biofilm exposed to QaC.

To assess *L. monocytogenes* susceptibility to QaC and BaC, LD_90_ values were calculated. Figure 6 presents QaC LD_90_ values. These values ranged from 298.0 to 532.2 ppm, and were higher than the manufacturer’s recommended concentrations to be used in food-related surfaces (maximum recommended concentration: 150 ppm). 

This fact is relevant, because QaC is a commercial biocide that might be used in sublethal concentrations, which might induce *L. monocytogenes* resistance [46,56]. *L. monocytogenes* QaC resistance has been previously described, both for planktonic cells and biofilms [10,22,35]. 

**Figure 7:**
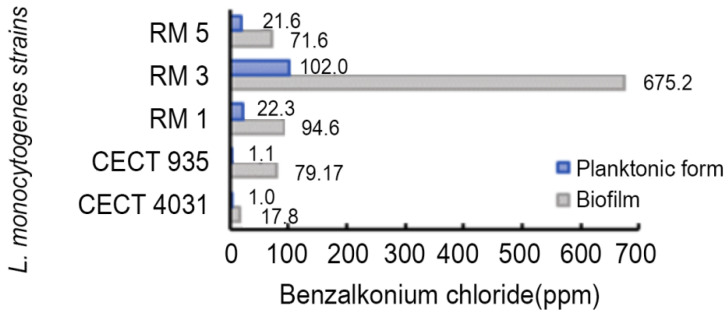
LD_90_ estimated values of *L. monocytogenes* tested strains in biofilm and in the planktonic form exposed to BaC.

BaC estimated LD_90_ values for *L. monocytogenes* tested strains (Figure 7) that ranged from 1.0 to 102.0 ppm in the planktonic form and from 17.8 to 675.2 ppm in biofilms, presenting significant differences (*p* < 0.0001). 

*L. monocytogenes* biofilms exhibited a reduced susceptibility to BaC, compared to the planktonic forms. The biofilm structure may play an important role as the extracellular matrix acts like a barrier, preventing contact with antimicrobial agents [57,58]. In this study, the higher the biofilm-forming ability, the higher were the LD_90_ values for QaC and BaC. This positive association of biofilm-forming ability and LD_90_ values was moderate, both for QaC and for BaC (Table 4). 

These results emphasize the importance of the cautious selection and use of sanitizers in food-producing premises. In fact, the equipment’s sanitizing method should be re-assessed and validated in order to control *L. monocytogenes* contamination, as it might select isolates that are able to survive and adapt to the food processing environment [59], acting as potential contamination sources for RTE food produced in those surfaces. Taken together, biofilm-forming ability and LD_90_ values underline the need to select different sanitizers, using rotating schemes, in order to prevent biocide resistance over time. Also, different strategies should be considered, other than the use of chemical biocides, as novel technologies, to control *L. monocytogenes* in the food production environment [60,61].

## 3. Materials and Methods 

### 3.1. Characterization of L. monocytogenes Isolates Collection

A collection of presumptive *L. monocytogenes* isolates (*n* = 20) was gathered from raw materials, intermediate, and final products, as well as industrial environment samples (food contact surfaces) of a RTE meat-based food producing industry (Table 1). This industry was located in Évora, Alentejo and produced pork meat delicatessens. *L. monocytogenes* isolates were collected during a 6-year period (2010–2015) as a result of routine microbiological sampling for industrial hygiene and food safety verification purposes, according to ISO 11290:1996 [62]. From a total of 76 collected samples, five raw materials, three intermediate products, nine finished RTE meat products and three food-contact surfaces were positive for *L. monocytogenes*. The strains were preserved in brain hearth infusion (BHI) broth (Scharlab, S.B., Barcelona, Spain) with 15% glycerol (Merck KGaA, Darmstadt, Germany) at −80 °C and revivified before use. 

### 3.2. L. monocytogenes Confirmation and Serogrouping by PCR

Presumptive *L. monocytogenes* isolates (*n* = 20) were confirmed by PCR and serogrouped using a multiplex PCR and an additional PCR based on the amplification of the *fla*A gene [27]. *L. monocytogenes* confirmed isolates (*n* = 17) were selected for further genetic characterization.

### 3.3. Pulsed-Field Electrophoresis Typing

PFGE typing of the selected isolates was performed according to the PulseNet standardized procedure for *L. monocytogenes* [63]. Briefly, bacterial genomic DNA in 1.5% agarose (SeaKem Gold Agarose, Cambrex, East Rutherford, NJ, USA) plugs was digested in separate reactions with 10U *Asc*I (NZYTech, Lisbon, Portugal) for 2h at 37 °C, and with 50U *Apa*I (NZYTech) for 2h at 25 °C. Electrophoresis of the resulting DNA fragments was performed in 1% agarose gel (SeaKem Gold), with a lambda PFG ladder standard (New England Biolabs, Massachusetts, USA) in 0.5 X solution of Tris–borate–EDTA buffer (NZYTech) at 14 °C, with 6 V/cm, initial pulsed time of 4.0 s and final pulsed time of 40 s, included angle of 120° over 19 h using a CHEF-Dr III System (Bio-Rad Laboratories, Hercules, CA, USA). Gels were stained with ethidium bromide (Sigma, St. Louis, MO, USA) and photographed under UV transillumination. 

### 3.4. Biofilm-Forming Ability Assay

To assess biofilm formation, six *L. monocytogenes* strains were selected (RM1, RM3, RM5, FP1, FP5, and FP8) to have representatives from different serogroups and PFGE types. Also, four *L. monocytogenes* reference strains from the Spanish Type Culture Collection (CECT) were used: CECT 4031 (serogroup IIa), CECT 937 (serogroup IIb), CECT 911 (serogroup IIc), and CECT 935 (serogroup IVb). These strains present the same serogroups as the ones detected in this study isolates (Table 2), allowing for the comparison with existing studies. 

The protocol proposed by Romanova et al. [51] was used with some modifications to obtain a 5-day *L. monocytogenes* mono-cultural biofilm. A single colony of each selected strain was inoculated in buffered peptone water (BPW) (Scharlab, S.B) and incubated for 16–18 h at 30 °C. Optical density at 600 nm (OD_600nm_) was assessed in Ultrospec 2000 (Pharmacia Biotech, Washington, WA, USA) to obtain a concentration of 8 log cfu/mL. For each strain, 4 μL were transferred into three separate wells of polystyrene flat-bottomed microtiter plates (Normax, Marinha Grande, Portugal) filled with 200 μL of BPW. Three wells were used as negative controls, with BPW alone. The plates were lidded and statically incubated at 30 °C for 5 days. After this, the solution was removed from the wells that were rinsed once with sterile distilled water to remove loosely associated bacteria and the attached biofilms were assessed by viable cells enumeration and crystal violet staining. 

Considering both evaluation methods, this assay was performed in triplicate, with three replicates for each strain.

#### 3.4.1. Enumeration of Viable Cells in Biofilms 

The biofilm was detached from the well surface mechanically into 100 μL of BPW using a mini cell scraper (VWR International, Monroeville, PA, USA). The microtiter plate was sonicated (Ultrasonic bath MXB14, Grant Instruments, Royston, England) for 5 min to detach and collect sessile cells. Then, 100 μL of BPW were pipetted into each well. Serial 10-fold dilutions of the sample in BPW were prepared and 10 μL were dropped onto the surface of a tryptone soy agar (TSA) (Scharlab, S.B) plate. After overnight incubation at 30 °C, colonies were enumerated in a stereoscopic magnifier (Nikon SMZ645, Tokyo, Japan).

#### 3.4.2. Biofilm Assessment by Crystal Violet Staining

The microtiter plate was left air drying for 45 min in the laminar flow hood. Biofilm was stained by adding 220 μL of 0.1% crystal violet (bioMérieux, France) solution to each well for 15 min at room temperature. After stain removal, the wells were washed three times with sterile distilled water and left air drying for 30 min in the laminar flow hood. Then, 220 µL of detaining solution (ethanol: acetone 80:20 *v/v*) were added to each well 15 min. The microtiter plate was then shaken (Ultrasonic bath MXB14, Grant Instruments, Royston, England) for 5 min and the crystal violet OD (cvOD) was measured in SpectraMax 340PC (Molecular Devices, Silicon Valley, San Jose, CA, USA). Each absorbance value was corrected by subtracting the average absorbance readings of the blank control wells. Adherence capability of the tested strains was based on the cvOD exhibited by bacterial biofilms, according to Stepanović et al. [40] classification. The cut-off cvOD (cvODc) was defined as three standard deviations above the negative control mean cvOD. Strains were classified as no biofilm producers (cvOD ≤ cvODc), weak biofilm producers (cvODc < cvOD ≤ 2 × cvODc), moderate biofilm producers (2 × cvODc < cvOD ≤ 4 × cvODc), and strong biofilm producers (4 × cvODc < cvOD). 

### 3.5. Biocides Activity Testing Assay

Based on serogrouping and biofilm formation parameters, *L. monocytogenes* strains RM1, RM3 and RM5 and *L. monocytogenes* reference strains CECT 4031 and CECT 935 were selected to be further assessed.

Biocides activity testing was performed according to European standard EN 1276:2009 [64], using the quantitative suspension test for bactericidal activity evaluation of chemical disinfectants used in food and industrial areas. To simulate clean conditions, in all tests, 0.03 g/L of bovine serum albumin (Merck KGaA) was used as an interfering substance. Contact time (5 min) and temperature (20 °C) were established according to the obligatory test conditions specified in EN 1276:2009. For all strains, experimental conditions were previously validated. Biocide activity was assessed using *Escherichia coli* DSMZ 682, *Pseudomonas aeruginosa* ATCC 15442, *Staphylococcus aureus* CECT 239, and *Enterococcus hirae* ATCC 10541D-5, to validate dilution-neutralization, absence of lethal effect in test conditions, including neutralizer toxicity, and efficacy of neutralizing solutions. 

Commonly used biocides in food contact surfaces and equipment sanitization in food-producing establishments were selected for further testing. Commercial sanitizers (HigiaBlue, Portugal) containing oxidizing compounds (OxC), ethanol-based compounds (EthC) and quaternary ammonium compounds (QaC) were tested. Benzalkonium chloride (BaC; Merck KGaA) was also evaluated. Table 5 exhibits the tested concentrations for each biocide (diluted in hard water) and respective neutralizers. 

All measurements were performed in duplicate and all experiments were performed twice.

#### 3.5.1. Activity towards *L. monocytogenes* Planktonic Suspension

*L. monocytogenes* strains were incubated in BHI agar (Scharlab, S.B.) at 37 °C for 18 h. Then, 10 mL of the bacterial suspension were prepared to have an OD_600nm_ of 0.15–0.5, corresponding to a concentration of approximately 1.5–5 × 10^8^ cfu/mL. To each tube containing 1 mL of interfering substance, 1 mL of the bacterial suspension was added, and the mixture was vortexed. After 2 min, 8 mL of one of the desired biocide test concentration were added, incubating for 5 min at 20 °C. Then, 1 mL was collected and mixed with 1 mL of hard water and 8 mL of the appropriate neutralizer. After neutralization (5 min at 20 °C), 1 mL was incorporated in TSA (Scharlab, S.B.) in duplicate. After overnight incubation at 37 °C, colonies were enumerated.

#### 3.5.2. Activity towards *L. monocytogenes* 5-day-old Biofilms

For the biocide activity testing on *L. monocytogenes* 5-day-old biofilms, to each well containing the biofilm, 20 μL of interfering substance and 20 μL of tryptone salt solution (Scharlab, S.B.) were added. After 2 min, 160 μL of one of the biocide test concentrations was added and incubated for 5 min at 20 °C. After medium removal, the wells were washed with 40 μL of hard water and 160 μL of the appropriate neutralizer. After neutralization (5 min at 20 °C), the medium was removed and the wells were washed with sterile distilled water, which was also removed. The biofilm quantification was performed according to the procedure described in Section 3.4.1. for biofilm detachment, dilution and colony enumeration. 

LD_90_ was then calculated for both planktonic and biofilm assays in order to determine the biocide concentration that reduced 90% of VCC.

### 3.6. Data Analyses

All quantitative data are presented as mean values with standard deviation (SD) from three independent experiments. Using BioNumerics software package version 6.10 (Applied Maths, Sint-Martens-Latem, Belgium), a dendrogram was constructed based on PFGE patterns of the 17 *L. monocytogenes* strains, with an optimization setting of 1.5% and a band-position tolerance of 1.5% for *Asc*I and *Apa*I restriction. Cluster analysis was performed using the unweighted pair group method (UPGMA) with arithmetic averages and band-based Dice correlation coefficient.

To assess *L. monocytogenes* biofilm formation parameters, Pearson’s correlation analyses were used to evaluate the interdependency of cvOD and VCC. To relate biofilm formation parameters and *L. monocytogenes* serogroups, one-way analysis of variance (ANOVA) followed by Tukey’s test were performed.

To evaluate the susceptibility of selected *L. monocytogenes* strains to biocides, LD_90_ values were obtained by adjusting experimental data of mortality obtained in biocide testing assays to a polynomial equation or to a linear regression adjusted to a scatter plot of mortality versus biocide concentration in MS Excel 2016 software (Microsoft Corporation, Redmond, USA). Two-way ANOVA was used to compare BaC LD_90_ values in planktonic and biofilm forms. To compare *L. monocytogenes* biofilms QaC LD_90_ values and also BaC LD_90_ values, one-way ANOVA followed by Tukey’s test were performed. Pearson’s correlation coefficient was also used to relate biofilm formation parameters and QaC and BaC LD_90_. When *p* < 0.05, a statistically significant difference was considered.

## 4. Conclusions

Overall, this study provided an assessment of *L. monocytogenes* isolates from a RTE meat-based food industry, using phenotypic and genetic characterization. The use of molecular and subtyping techniques is an important tool to understand the routes and sources of contamination. Our results reveal that *L. monocytogenes* contamination of finished products seems to be related to food-contact surfaces, but also to raw materials. Moreover, some of the obtained pulsotypes revealed high homology (>90%) but were not temporally matched, being collected with months of interval. These results might point out to a common source of contamination and are consistent with the hypothesis that there are stable clonal groups of *L. monocytogenes*, which persist over time, in foods and food-related environments.

All of the studied *L. monocytogenes* strains demonstrated biofilm-forming ability at 30 °C, revealing to be weak biofilm producers. Strains in biofilms were not susceptible to one of the used commercial sanitizers in the industrial premises, QaC, within the recommended concentration range. Similar results were obtained when testing a pure substance biocide, benzalkonium chloride (BaC) in *L. monocytogenes* biofilms. In contrast, *L. monocytogenes* planktonic forms were susceptible to BaC tested concentrations. A positive association was found between biofilm formation parameters and LD_90_ values for QaC and BaC.

Taken together, our results suggest that preventive measures need improvement in the assessed food industry. It also reinforces the necessity of an appropriate selection and application of biocides in food premises, to prevent *L. monocytogenes* biofilm formation and biocide resistance development over time.

## Figures and Tables

**Table 1:** Samples collected in the assessed industry with positive *L. monocytogenes* detection by conventional microbiological methods.

Date of Collection	Sample Description/Type	Presumptive *L. monocytogenes* Isolate Code
July 2010	*Chourição*/Final product	FP1
February 2013	*Chouriço*/Final product	FP2
March 2013	Seasoned ham meat/Intermediate product	IP1
March 2013	In-use meat mincing machine/Equipment	E1
March 2013	Meat sausage/Final product	FP3
April 2013	Unseasoned ham meat/Intermediate product	IP2
April 2013	Seasoned ham meat/Intermediate product	IP3
April 2013	Pork meat/Raw material	RM1
April 2013	In-use meat mincing machine/Equipment	E2
April 2013	Raw meat transport box/Equipment	E3
July 2013	Pork meat/Raw material	RM2
October 2013	*Chouriço*/Final product	FP4
February 2014	Lard for *chouriço*/Raw material	RM3
February 2014	*Chouriço*/Final product	FP5
April 2014	Boneless pork shoulder/Raw material	RM4
May 2014	*Chouriço*/Final product	FP6
May 2014	*Alheira*/Final product	FP7
January 2015	Boneless pork shoulder/Raw material	RM5
February 2015	*Farinheira*/Final product	FP8
April 2015	*Chouriço*/Final product	FP9

**Table 2:** Description of the obtained serogroups among *L. monocytogenes* confirmed isolates (*n* = 17).

Serogroup	Proportion	Isolate Code ^1^
IIa	6 (35.3%)	FP1, RM2, RM3, FP7, FP8, FP9
IIb	1 (5.9%)	FP5
IIc	9 (52.9%)	FP2, IP1, E1, FP3, IP2, RM1, E2, E3, RM4
IVb	1 (5.9%)	RM5

^1^*L. monocytogenes* isolates share the same code with the sample from which they were recovered.

**Table 3:** Biofilm-forming ability of *L. monocytogenes* strains according to the respective serogroups.

*L. monocytogenes* Serogroup	*n*	Log cfu/mL (Mean ± SD)	cvOD (Mean ± SD)
IIa	4	7.2 ± 0.8	0.096 ± 0.019
IIb	2	7.0 ± 0.4	0.114 ± 0.002
IIc	2	7.0 ± 0.7	0.099 ± 0.003
IVb	2	7.1 ± 0.5	0.107 ± 0.001

**Table 4:** Pearson’s correlation coefficients for biofilm-forming ability parameters and LD_90_ values for QaC and BaC.

LD_90_ Values	Log cfu/mL	QaC LD_90_
QaC	0.652	1
BaC	0.554	0.607

**Table 5:** Tested biocides, concentration range and neutralizers used in biocide activity testing assay (EN 1276:2009).

Biocide	Tested Concentrations			Neutralizer
Oxidizing compound (OxC)	50 ppm	100 ppm	150 ppm	Polysorbate 80, 30 g/L + lecithin, 3 g/L + sodium thiosulphate 10 g/L
Quaternary ammonium compound (QaC)	50 ppm	100 ppm	150 ppm	Polysorbate 80, 30 g/L + sodium dodecyl sulphate, 4 g/L + lecithin, 3 g/L
Ethanol-based compound (EthC)	50%	70%	100%	Polysorbate 80, 30 g/L + saponin, 30 g/L + lecithin, 3 g/L
Benzalkonium chloride (BaC)	Planktonic cells		Biofilm	
	0.8–150 ppm		0.2–500 ppm	Polysorbate 80, 30 g/L + sodium dodecyl sulphate, 4 g/L + lecithin, 3 g/L
